# Development and characterization of penta-flowering and triple-flowering genotypes in garden pea (*Pisum sativum* L. var. *hortense*)

**DOI:** 10.1371/journal.pone.0201235

**Published:** 2018-07-30

**Authors:** Jyoti Devi, Gyan P. Mishra, Satish K. Sanwal, Rakesh K. Dubey, Prabhakar M. Singh, Bijendra Singh

**Affiliations:** 1 ICAR-Indian Institute of Vegetable Research, Varanasi, India; 2 ICAR-Indian Agricultural Research Institute, Pusa, New Delhi, India; 3 ICAR- Central Soil Salinity Research Institute, Karnal, India; National Bureau of Plant Genetic Resources, Pusa India, INDIA

## Abstract

This study reports the development of a garden pea genotype ‘VRPM–901–5’ producing five flowers per peduncle at multiple flowering nodes, by using single plant selection approach from a cross ‘VL-8 × PC-531’. In addition, five other stable genetic stocks, namely VRPM-501, VRPM–502, VRPM–503, VRPM–901–3 and VRPSeL–1 producing three flowers per peduncle at multiple flowering nodes were also developed. All these unique genotypes were of either mid- or late- maturity groups. Furthermore, these multi-flowering genotypes were identified during later generations (F_4_ onward), which might be because of fixation of certain *QTLs* or recessive gene combinations. Surprisingly, a common parent PC–531, imparting multi-flowering trait in ten cross combinations was identified. Thus, the genotype PC–531 seems to harbor some recessive gene(s) or *QTLs* that in certain combination(s) express the multi-flowering trait. The interaction between genotype and environment showed that temperature (11–20°C) plays a key role in expression of the multi-flowering trait besides genetic background. Furthermore, the possible relationship between various multi-flowering regulatory genes such as *FN*, *FNA*, *NEPTUNE*, *SN*, *DNE*, *HR* and environmental factors was also explored, and a comprehensive model explaining the multi-flowering trait in garden pea is proposed.

## Introduction

The garden pea (*Pisum sativum* L. 2n = 2x = 14) is one of the first genetic model legumes that was used to understand the basic concepts of genetics [1]. World production of green peas in 2016 was 19.88 mt, and the major producers were China (12.21 mt), India (4.81 mt), and USA (0.31 mt), which accounted for >85% of the total production [2]. Peas are quite inexpensive and a readily available source of proteins, vitamins, minerals, and carbohydrates, making them a valuable food capable of meeting the global dietary needs of >900 million undernourished people [3]. The floral and inflorescence morphology of pea has drawn the attention of researchers, who described it as compound indeterminate inflorescence [4–8]. The shoot apical meristem, after the transition to flowering (referred to as I_1_), produces lateral compound leaves with the pairs of leaflets and tendrils, bearing an axillary inflorescence meristem (referred to as I_2_). Once flowering is initiated, the first node is termed as the node of flower initiation (NFI), whereas the subsequent ones are called floral nodes. Besides fundamental interest, the regulations of inflorescence development in pea are of special practical value for breeding of novel highly productive cultivars [9]. Inflorescence traits such as the total number of flowering nodes per plant and the number of flowers per node are amenable to improvement in legumes, as these traits directly determine the number of pods and yield per plant [10].

The number of flowers per raceme or peduncle is a genetically determined character, which may vary from one to many [11, 12]. With very limited variation in the flower number per node, most garden pea varieties/germplasm lines generally have a single or double flower per node [13, 14] with more single-flower panicles at the upper nodes [15]. The development of three or more pods per node, while maintaining the pod size over one and two, appears an attractive option to increase the yield [16], besides increasing the number of effective ovules per pod. However, the use of these traits in the yield improvement is limited because of the lack of precise information about the regulation and stability of these traits.

Multi-flowering racemes, alternatively referred to as many-flowered raceme or multi-podded phenotype, have been reported in related species such as *Pisum elatius*, bearing 2–3 flowers per peduncle [17], and *Pisum arvense*, bearing three or more flowers per peduncle [18]. A close relation between pea and other Fabeae species, exhibiting a wide range of variation in the number of flowers per node, may have different mechanisms for the regulation of flower numbers per peduncle [19, 20]. In India, VRP–500 (INGR15009) was the first multi-flowering genetic stock reported to have three flowers per peduncle at multiple flowering racemes [21].

Despite its possible influence on pod and seed yield, limited attention has been given to the improvement of this complex multi-flowering trait. Although various garden pea genotypes producing three or more flowers per I_2_ have been constantly reported [22–32], its regulation has not yet been properly deciphered, especially in different backgrounds. In this backdrop, the present study aimed to develop and characterize various multi-flowering genotypes bearing three to five flowers on a single peduncle.

## Materials and methods

During four-year study period viz. winter-season of the year 2014–15, 2015–16, 2016–17 and 2017–18, a total of 583 genotypes including 450 germplasm lines, 30 cultivated varieties, and 103 advanced breeding lines were grown in our experimental fields. These genotypes were raised under normal field conditions following standard cultural practices. The plants were grown at a row-to-row and plant-to-plant spacing of 10 × 30 cm in a bed size of 3 × 3 m, occupying 300 plants per plot. The study was conducted at the research farm of Indian Institute of Vegetable Research, Varanasi, India, which is located at 82°52ʹ37ʹʹ E and 25°18ʹ21ʹʹ N at an elevation of 83 m above the mean sea level (AMSL). The detailed experiment methodology involving different parents and crosses to develop multi-flower genotypes in garden peas is presented in S1 Fig.

### Data recording

The plants were categorized as one or two flowering types and multi-flowering types (when any of the axillary racemes produced more than two flowers on a single peduncle). Based on the days taken to 50% flowering, the genotypes were also grouped as early (<40 d), mid (40 to 60 d), and late (>60 d) maturity groups. Nine genotypes were intensively studied, of which six (VRP–500, VRPM–501, VRPM–502, VRPM–503, VRPM–901–3, and VRPSeL–1) were of three flowers per peduncle type, one (VRPM–901–5) was of five flowers per peduncle type, while other two were commercially grown cultivars (PC–531 and VL–8) forming two flowers per peduncle.

The observations were recorded on 14 traits, namely days to first flowering (DFF), appearance of first multi-flowering node (AFMFN; in the multi-flowering phenotype), number of branches per plant (BPP; No), peduncle length (PL; cm), peduncle diameter (PD; cm), pod length (PL; cm), pod width (PW; cm), average pod weight (APW; g), number of pods per plant (PPP), number of seeds per pod (SPP), plant height (PH; cm), total number of flower(s) produced (TFP), percentage conversion of multi-flower to multi-pods (PCTMP,%), and yield per plant (YPP; g). In addition, observations were also made for 15 plants of the penta-flowering genotype (VRPM–901–5) for nine inflorescence-related traits, namely first flowering/fertile node (FFN), number of nodes producing one, two, three, four, and five flowers per peduncle (NON), number of nodes bearing five-flowered racemes, total number of flowers produced (TFP), and total number of reproductive nodes (TRN). The nodes were counted by assuming the scale leaves as node one.

### Weather parameters

Various meteorological data, such as minimum, maximum, and average temperature (°C); high and low humidity (%), wind speed (km/h), precipitation (mm), and day length (h) were taken for four consecutive cropping seasons during November to April months of the year 2014–15 till 2017–18, from the meteorological observatory located at ICAR-Indian Institute of Vegetable Research, Varanasi, India (S2–S5 Figs).

### Statistical analysis

The mean, standard deviation, standard error, and coefficient of variation (CV) of each trait were calculated by subjecting the data on yield and related component traits to the analysis of variance [33]. Furthermore, genotypic and phenotypic correlations were also calculated [34]. Multiple comparisons of the mean were done using Tukey-Kramer’s Honest Significant Difference test (p<0.05). All statistical analyses were performed using Windostat version 8.5 (http://www.indostat.org).

## Results and discussion

### Development of multi-flowering genotypes

**Fig 1:**
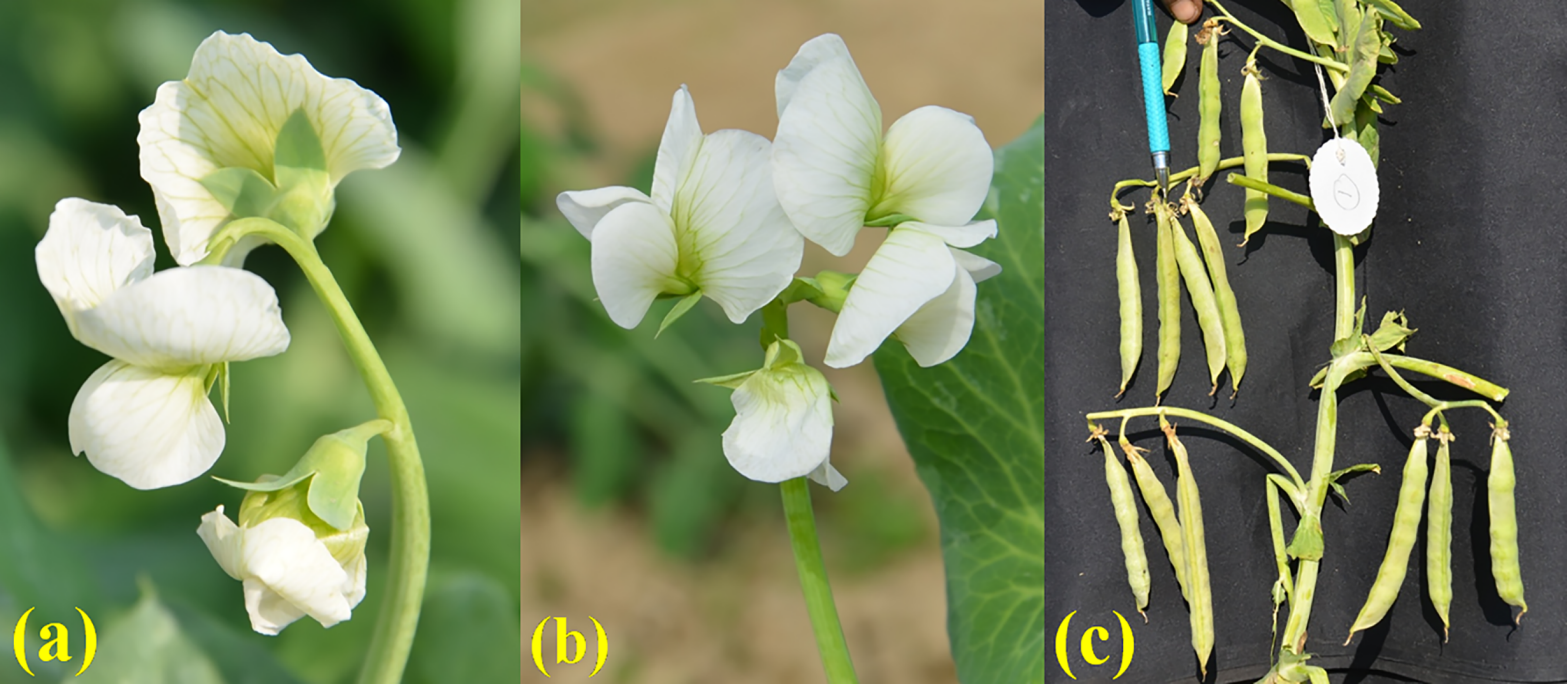
Multi-flowering lines. (a) three flowers per peduncle; (b) four flowers per peduncle; and (c) three and four pods per peduncle.

**Fig 2:**
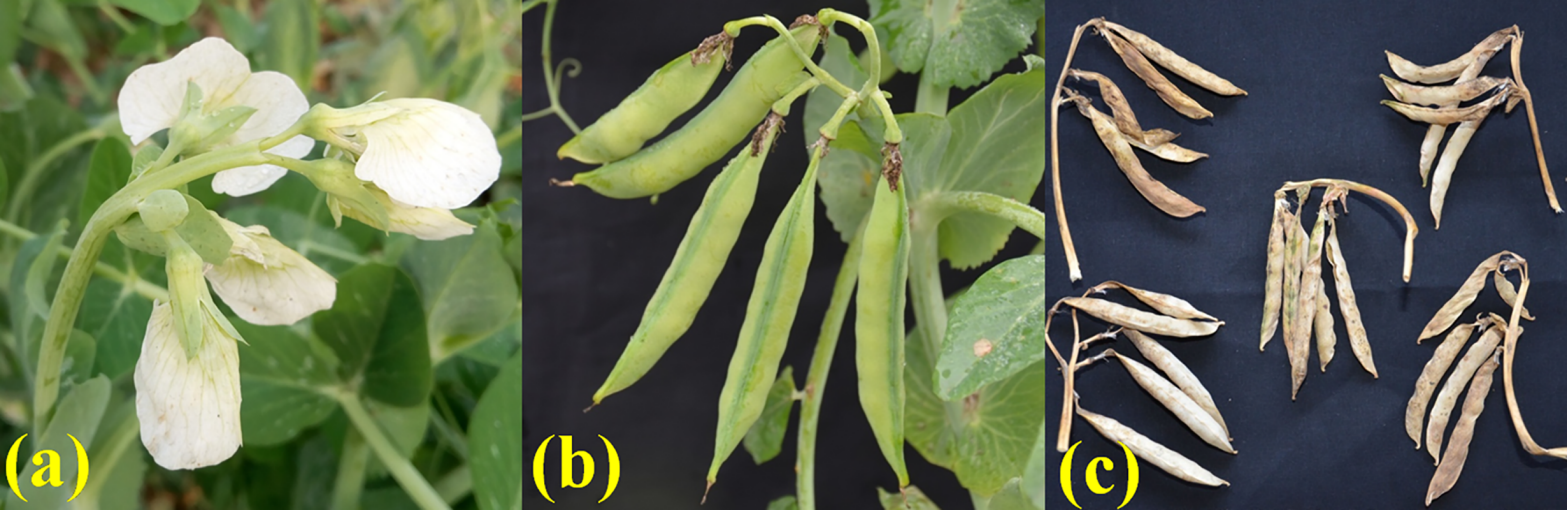
VRPM–901–5 genotype. (a) five flowers per peduncle and (b-c) five pods per peduncle in garden pea.

A total of six, stable multi-flowering garden pea genetic stocks were developed, of which five, namely VRPM–501, VRPM–502, VRPM–503, VRPM–901–3, and VRPSeL–1 (a selection from *Shihara LocaL–1*) produced three flowers per peduncle at multiple flowering nodes (Fig 1), while VRPM–901–5 produced five-flowers per peduncle at multiple flowering nodes (Fig 2). In addition, VRP–500 (INGR15009) was the first stable triple-flowered genetic stock, earlier developed at Indian Institute of Vegetable Research, Varanasi, India [21]. All the genotypes reported in this investigation produced multi-flowering trait in every generation at multiple flowering nodes, but not on all the flowering nodes. Furthermore, based on our knowledge, no garden pea genotype from any part of the world is known to form multiple flowers on all flowering nodes. The comparative details of most multi-flowering garden pea genotypes including genotypes used in this study are summarized in Table 1.

**Table 1:** Details of the multi-flowering garden pea genotypes across the world.

S. No.	Genotypes	3 Flowers per node	4 Flowers per node	5 Flowers per node	Generation/remarks	Pods per peduncle	Stability	Reference
1.	Mangetout Tardif cossejaune×Pois sans parcheminbeurre	√	√	√*	F_4_	Not Reported	Not reported	23
2.	Unnamed	**√**	-	-	-	-	-	24
3.	Unnamed	**√**	×	×	-	Reported	Stable	26, 27
4.	Daehnfeldt multi podded selection	**√**	×	×	-	Not reported	Not reported	43
5.	Puget, Sleaford Orbiter	√	√	√*	Multi-flowered varieties grown under controlled conditions	Not reported	Stable up to 4 flowers	15
6.	Trio, JTPGRO 8, Puget Recette, Neptune, Clamart a riosgousses, JI382, Sleaford Orbiter Martus	√	√	√*	Multi-flowered varieties Grown under controlled conditions	Not reported	Not stable	30
7.	Dark Skinned Perfection	√	×	×	Selection within variety	Not reported	Not reported	31
8.	Induced mutants	√	√	√*	-	Reported	Not reported	32
9.	Induced mutant *nep–1* and *nep–2*	√	√	√*	-	Reported	Not reported	45
10.	VRP–500 (VRP–5× PC–531)	√	×	×	F_6_	Reported	Stable	21
11.	Lupinoid×DTR	√	×	×	F_2_	Not reported	-	9
12.	VRPM–901–5 (VL–8×PC–531)	√	√	√	F_5_	3–5 pods	Stable	This study
13.	VRPM–501 (PC–531×Pusa Pragati)	√	×	×	F_5_ to F_8_	2–3 pods	Stable	This study
14.	VRPM–502 (VRP–186×VRP–500)	√	×	×	F_5_ to F_8_	2–3 pods	Stable	This study
15.	VRPM–503 (Arkel×Azad Pea–3)	√	×	×	F_5_ to F_8_	2–3 pods	Stable	This study
16.	VRPM–901–3 (VL–8×PC–531)	√	×	×	F_5_ to F_8_	2–3 pods	Stable	This study

*Only occasional appearance of five flowers per peduncle was reported.

### VRPM–901–5: A five-flowers per peduncle genotype

During the winter-season of 2015–16, a plant producing five flowers per peduncle at some floral nodes was identified in the F_4_ generation (VL–8×PC–531), which was named as VRPM–901–5. In addition, two more plants having four flowers at some of the peduncles were also identified. Interestingly, both the parents involved in the crossing produced only two flowers per node. The detailed characteristics of the parental lines are presented in Table 2. The seeds from these plants harvested as single plant progenies (SPS) were advanced to the F_5_ generation, which was grown in the winter-season of 2016–17, again produced five flowers per peduncle on certain buds. Interestingly, five-flowered racemes were also observed in some of the progenies of plants that produced four-flowered racemes during the winter-season of 2015–16.

**Table 2:** Mean comparison of new multi-flowering genotypes along with double-flowering cultivars through Tukey-Kramer’s HSD test.

^1^Traits/ Genotypes	DFF	AFMFN	BPP	PDL	PDD	PL	PW	APW	PPP	SPP	PH	TFP	PCTMP	YPP
**VRP–500**	49.3^c^	16.0^b^	1.72^ab^	5.93^f^	0.23^a^	7.50^abc^	1.43^b^	5.97^ab^	30.33^abc^	7.00^a^	84.0^bcd^	34.66^c^	66.17^a^	160.0^abc^
**VRPM–501**	49.0^c^	13.0^b^	1.67^ab^	13.90^a^	0.23^a^	9.60^a^	1.20^bc^	5.67^abc^	28.10^abcd^	6.50^a^	110.6^ab^	33.7^c^	75.67^a^	145.0^abcd^
**VRPM–502**	52.0^bc^	13.0^b^	2.00^a^	7.17d^cde^	0.20^ab^	7.80^ab^	1.44^b^	4.43^cd^	27.27^bcd^	6.27^ab^	73.3^d^	33.3^cd^	62.33^a^	115.0^cd^
**VRPM–503**	57.3^b^	16.3^b^	1.33^b^	8.25b^bc^	0.23^a^	8.27^ab^	1.23^bc^	6.00^ab^	26.77^cd^	6.00^ab^	103.8^abc^	33.0^cd^	75.00^a^	146.7^abc^
**VRPM–901–3**	48.7^c^	13.0^b^	1.93^a^	7.57^bcd^	0.22^a^	9.10^a^	1.20^bc^	5.50^bc^	36.83^ab^	6.87^a^	115.0^a^	44.67^ab^	71.00^a^	181.3^a^
***VRPSeL–1***	65.0^a^	14.0^b^	2.00^a^	6.15^ef^	0.14^b^	5.50^c^	1.10^bc^	3.60^d^	33.33^abc^	4.83^b^	81.4^cd^	50.67^a^	16.33^b^	91.7^d^
**VRPM–901–5**	49.5^c^	21.9^a^	2.13^a^	6.72^def^	0.22^a^	7.77^ab^	1.00^c^	5.36^bc^	37.67^a^	4.81^b^	124.1^a^	50.2^a^	64.00^a^	177.4^ab^
PC–531*	51.0^bc^	*0.0^c^	1.34^b^	8.57^b^	0.20^ab^	9.19^a^	1.83^a^	7.05^a^	20.00^d^	6.67^a^	66.3^d^	23.7^d^	0.00^b^	113.5^cd^
VL–8*	69.0^a^	*0.0^c^	1.20^b^	5.67^f^	0.20^ab^	6.10^bc^	1.14^bc^	5.00^bcd^	30.33^abc^	6.67^a^	115.2^a^	37.7^bc^	0.00^b^	125.0^bcd^
**Mean ±SE**	54.5 ±1.38	11.9 ±0.83	1.7 ±0.11	7.8 ±0.24	0.2 ±0.013	7.9 ±0.45	1.3 ±0.07	5.4 ±0.29	30.1 ±1.99	6.2 ±0.31	97.1 ±5.81	37.9 ±2.00	47.83 ±3.80	139.51 ±10.94
**R**^**2**^	**0.93**	**0.97**	**0.82**	**0.98**	**0.70**	**0.81**	**0.83**	**0.84**	**0.77**	**0.77**	**0.85**	**0.90**	**0.97**	**0.78**
**CD (5%)**	**3.20**	**2.18**	**0.11**	**0.61**	**0.03**	**1.33**	**0.24**	**0.74**	**5.24**	**0.95**	**16.74**	**5.28**	**8.92**	**24.93**

Values followed by the same letter in each column are not significantly different by Tukey-Kramer’s HSD test (p < 0.05)

^1^DFF: Days to first flowering; AFMFN: Appearance of first multi-flowering node; BPP: No. of branches per plant; PDL: Peduncle length (cm); PDD: Peduncle diameter (cm); PL: Pod length (cm); PW: Pod width (cm); APW: Average pod weight (g); PPP: No. of pods per plant; SPP: No of seeds per pod; PH: Plant height (cm); TFP: Total no. of flower produced; PCTMP: Percent conversion to multi-pods; YPP: Yield per plant (g)

*Multi-flowering nodes were not present in the genotypes PC–531 and VL–8.

**Fig 3:**
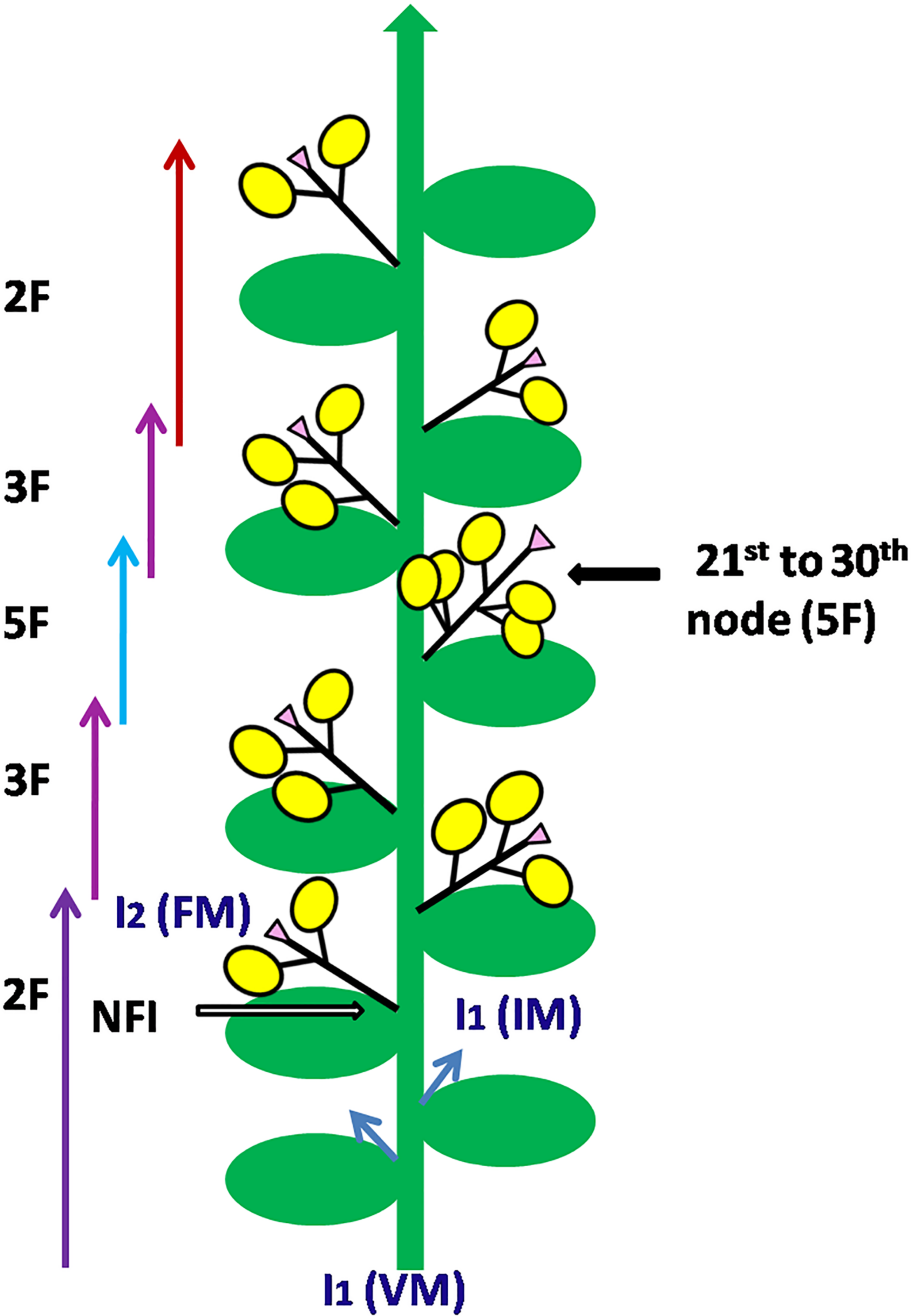
Pictorial presentation of a compound raceme of the garden pea, resulting in two, three and five flowers per peduncle at specific nodes. Green arrows indicate indeterminate growth of the inflorescence stem (I; I1); Green ovals represent leaves; Yellow circles represent flowers; Pink triangles are stubs terminating at each secondary inflorescence (I_2_) axis; VM: Vegetative meristem; IM: Inflorescence meristem; I1M: Primary inflorescence meristem; I_2_M: Secondary inflorescence meristem; FM: Floral meristem; NFI: First node of flower initiation.

Pea normally has the racemose type of inflorescence, and at the individual axis, only one or two flowers are borne. However, VRPM–901–5 showed a sequential appearance of flowers bearing up to five flowers per peduncle arranged in a compound inflorescence (Fig 3). Genetic control of multi-flowering was reported in chickpea [8, 35] and a recessive gene (*cym*) responsible for cymose inflorescence was identified [19]. The detailed characterization of various inflorescence-related traits was also done on 15 plants of VRPM–901–5 (S1 Table). The first flowering raceme appeared from the 19^th^ node (mostly 2–3 flowers per peduncle) and flowering was observed to continue till the 33^rd^ node (mostly 1–2 flowers per peduncle). Out of 284 fertile nodes examined, 128 had three flowers per peduncle, one had four flowers per peduncle, and 24 had five flowers per peduncle. Interestingly, most of the penta-flowering racemes appeared after the 22^nd^ node, indicating that this trait also gets influenced by environmental factors.

Out of 284 fertile nodes examined, 153 (53.87%) showed the multi-flowering trait, depicting a very high frequency, compared with only 0.17% reported earlier [31]. Furthermore, the expression of five flowers per peduncle for three consecutive years (2015–16, 2016–17 and 2017–18) suggested that the complex genetic factor(s) are regulating this complex trait, as it appeared not before F_4_ generation. In contrast, to the best of our knowledge, none of the earlier reports confirmed the stability of this trait in any genotype. Moreover, most of the multi-flowering genotypes reported in the literature are the result of a spontaneous mutation, which did not show stability in its expression (Table 1).

### Characterization of multi-flowering genotypes

The analysis of variance among nine garden pea genotypes showed highly significant differences (*P* ≤ 0.01) for all the 14 traits studied (S2 Table), indicating a high genetic variability among the genotypes. The appearance of first multi-flowering node did not differ significantly in all the multi-flowering lines. Profuse branching is another trait which was reported associated with multi-flowering [32, 36], and four of the seven multi-flowering genotypes studied showed prolific branching and were found significantly superior to double-podded cultivars.

The yield of a cultivar is dependent on the number of flowers produced and percentage conversion of these flowers to pods [37]. Our study showed that all the genotypes produced a significantly higher number of flowers than the double podded cultivar PC–531. However, shedding of flowers is common because of multiple reasons such as the genotypic background, nutritional factors, and adverse environmental conditions, resulting in reduced yield, especially in multi-podded cultivars [15, 16]. Although VRPSeL–1 produced maximum number of multi-flowers per plant (50.67), but the percentage conversion of multi-flowers to multi-pods was recorded least (16.33%) over other multi-flowering genotypes studied. This could be due to the very high flower-drop on the multi-flowering floral nodes of VRPSeL–1. Further, peduncle diameter was recorded least (0.14 cm) in this line when compared to other multi-flowering genotypes viz., VRP–500, VRPM-501, VRPM-503, VRPM-901-3 and VRPM-901-5 which ranged from 0.22 cm to 0.23 cm. Thus, less flower abscission in the multi-flowering lines having thicker peduncle (more diameter) might leading to formation of more pods and ultimately increased yield per plant in these multi-flowering lines [38]. Furthermore, two multi-flowering genotypes, VRPM–901–3 and VRPM–901–5, appeared most promising for pod yield per plant and were significantly superior to the double-podded cultivar PC–531. This could be attributed to the production of more number of flower and pods and higher percentage conversion of multi-flowers to multi-pods (Table 2).

Besides, other multi-flowering genotypes, namely, VRP–500, VRPM–501, and VRPM–503, were similar to the released variety PC–531 for pod length and average pod weight, and they gave significantly higher pod yield per plant, which could be attributed to the higher percentage conversion of multi-flowers to multi-pods (66.17%, 75.67%, and 75.0%, respectively) (Table 2). Thus, conversion of multi-flowers per peduncle to multi-pods per peduncle appears one of the promising reasons for the higher yield recorded in these multi-flowering genotypes.

### Peduncle traits and their association with the conversion of multi-flowers to multi-pods

Although a large number of reports regarding multi-flowering expression in peas exist, much emphasis has not been given on the study of this trait because of poor stability and poor conversion of multi-flowers to multi-pods. Moreover, peduncle traits are known for their diverse roles in crop productivity [39] and the most crucial trait include the transportation of assimilates to the filling pods so as to provide mechanical support to the developing tissue [40, 41]. The highest yielding genotypes, VRPM–901–3, and VRPM–901–5 were found at par with VRPSeL–1 for total flower produced, percentage conversion of multi-flowers to multi-pods per peduncle was significantly low in VRPSeL–1.

The different multi-flowering to the multi-pod conversion ratios of these genotypes also showed significant differences in the peduncle thickness in these genotypes (Table 2, S6 Fig). Furthermore, a significant positive correlation was observed between peduncle diameter and percentage conversion to multi-pods (r = 0.659*** and 0.760***) both at phenotypic and genotypic levels (Table 3). Additionally, peduncle diameter significantly correlated with pod length (r = 0.695*** and 0.799***), average pod weight (r = 0.671*** and 0.762***), the number of seeds per pod (r = 0.530^**^ and 0.569**), and pod yield per plant (r = 0.785*** and 0.937***) both at phenotypic and genotypic levels. Thus, peduncle thickness in a multi-flowering genotype is directly related to the number of pods produced on the multi-flowering nodes. This may lead to a better flow of assimilates to the developing pods and seeds in thick peduncle genotypes than in thin peduncle genotypes, which, in turn, causes significantly less flower-drop in the thick peduncle genotypes [40]. Moreover, the increase in the number of flowers per plant negatively affected various pod traits such as pod length (r = –0.499* and –0.531**), pod width (r = –0.830*** and –0.851***), average pod weight (r = –0.674*** and –0.617***), and seeds per pod (r = –0.679*** and –0.788***), which could be attributed to the competition for nutritional resources. The details of various other traits affecting the multi-flowering phenotype are presented in Table 3.

**Table 3:** Phenotypic and genotypic correlation coefficients for inflorescence, pod yield and related traits in new multi-flowering genotypes along with double-flowering cultivars.

^1^Traits		DFF	AFMFN	BPP	PDL	PDD	PL	PW	APW	SPP	PH	TFP	PPP	PCTMP	YPP
**DFF**	**P**	1.000	–0.411	–0.389	–0.427	–0.644***	–0.809***	–0.316	–0.517*	–0.251	0.029	0.215	0.008	–0.648***	–0.596**
	**G**	1.000	–0.418	–0.432	–0.432	–0.731***	–0.867***	–0.318	–0.542**	–0.289	0.029	0.210	0.013	–0.658***	–0.621***
**AFMFN**	**P**		1.000	0.708***	0.014	0.223	0.035	–0.530**	–0.255	–0.505*	0.303	0.546**	0.590**	0.791***	0.505*
	**G**		1.000	0.746^***^	0.018	0.270	0.047	–0.545**	–0.255	–0.554**	0.306	0.559**	0.615***	0.796***	0.532*
**BPP**	**P**			1.000	–0.119	–0.199	–0.075	–0.370	–0.529**	–0.513*	0.048	0.674***	0.653***	0.425	0.223
	**G**			1.000	–0.127	–0.266	–0.081	–0.386	–0.579**	–0.578**	0.016	0.732***	0.722***	0.454	0.266
**PDL**	**P**				1.000	0.383	0.720***	0.089	0.331	0.190	0.131	–0.365	–0.325	0.358	0.087
	**G**				1.000	0.421	0.770***	0.090	0.334	0.204	0.142	–0.373	–0.348	0.359	0.085
**PDD**	**P**					1.000	0.695***	0.072	0.671***	0.530**	0.424	–0.370	–0.040	0.659***	0.785***
	**G**					1.000	0.799***	0.086	0.762***	0.569**	0.510*	0.406	–0.043	0.760***	0.937***
**PL**	**P**						1.000	0.400	0.716***	0.451	0.071	–0.499*	–0.307	0.486	0.471
	**G**						1.000	0.413	0.806***	0.530**	0.068	–0.531**	–0.309	0.521*	0.549^**^
**PW**	**P**							1.000	0.596**	0.542**	–0.782***	–0.830***	–0.822***	–0.294	–0.317
	**G**							1.000	0.633***	0.661***	–0.861***	–0.851***	–0.863***	–0.316	–0.338
**APW**	**P**								1.000	0.539**	–0.035	–0.674***	–0.507*	0.073	0.384
	**G**								1.000	0.538*	–0.088	–0.617***	–0.536**	0.075	0.385
**SPP**	**P**									1.000	–0.140	–0.679***	–0.416	0.046	0.181
	**G**									1.000	–0.132	–0.788***	–0.514*	0.055	0.192
**PH**	**P**										1.000	0.498*	0.658***	0.353	0.681***
	**G**										1.000	0.538**	0.729***	0.371	0.736***
**TFP**	**P**											1.000	0.969***	0.116	0.229
	**G**											1.000	0.978***	0.123	0.228
**PPP**	**P**												1.000	0.328	0.534**
	**G**												1.000	0.343	0.540**
**PCTMP**	**P**													1.000	0.680***
	**G**													1.000	0.701***
**YPP**	**P**														1.000
	**G**														1.000

^1^DFF: Days to first flowering; AFMFN: Appearance of first multi-flowering node; BPP: Branches per plant; PDL: Peduncle length (cm); PDD: Peduncle diameter (cm); PL: Pod length (cm); PW: Pod width (cm); APW: Average pod weight (g); PPP: Pods per plant; SPP: Seeds per pod; PH: Plant height (cm); TFP: Total flower produced; PCTMP: Percent conversion to multi-pods; YPP: Yield per plant (g); P: phenotypic correlation coefficient; G:genotypic correlation coefficient

*Significant at r = >0.01; the bold values represent significance levels if correlation r = >0.001 (***) and r = >0.005 (**).

### Inheritance of multi-flowering trait in *Pisum*

**Fig 4:**
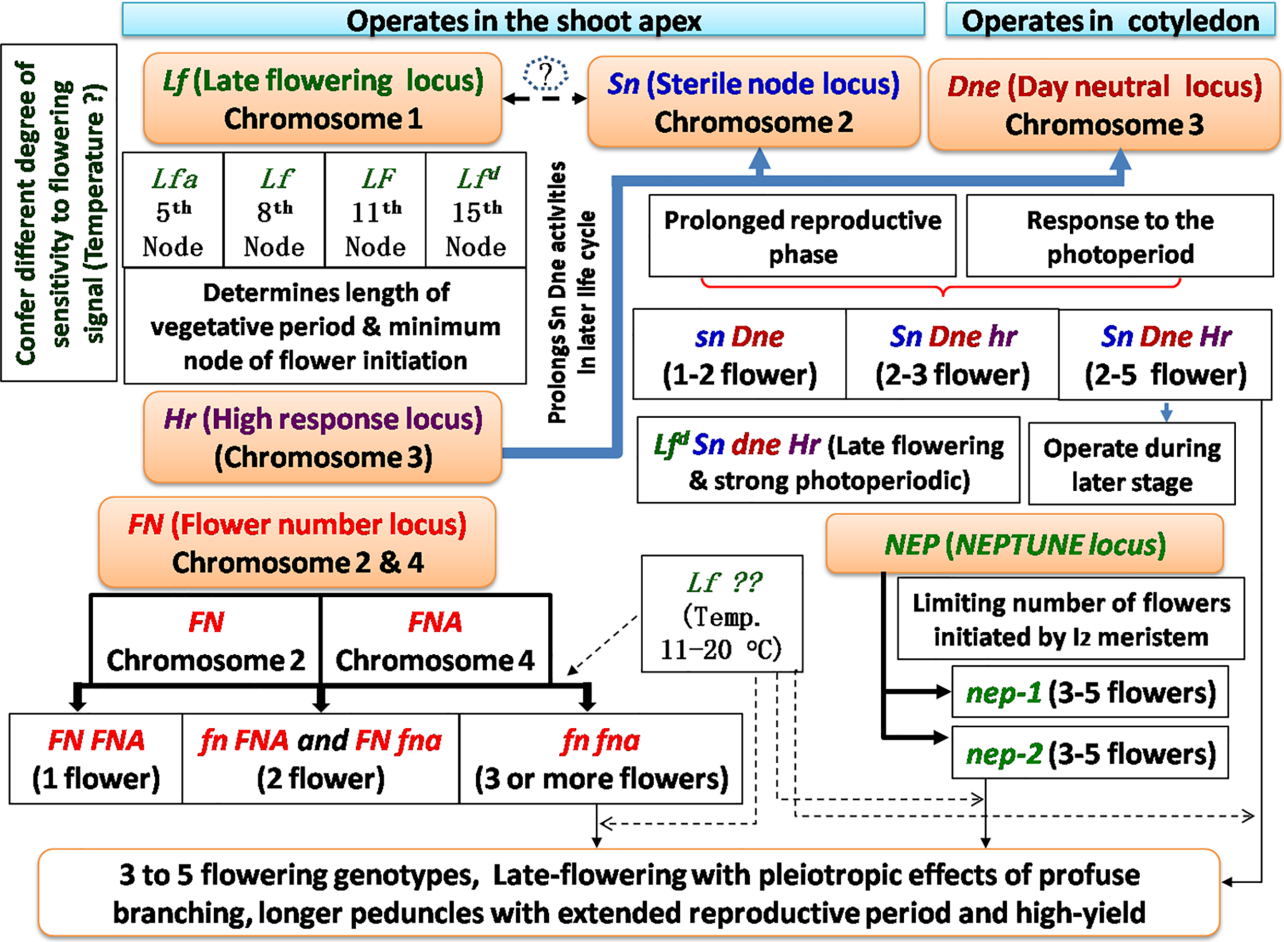
A comprehensive presentation of multi-flowering expression in garden pea. Integration of Lamprecht [24] (*FN*); Murfet [32] (*Sn*, *Hr*, *Dne*, and *LF*) and Singer et al. [45] (*nep*) gene models.

Although no population-based genetic analysis so far has reported for the multi-flowering trait in different known multi-flowering garden pea genotypes, various researchers have proposed multiple genes or polygenes controlling this trait [15, 26]. Among the 12 crosses studied, the multi-flowering trait was recorded in F_4_ (8 genotypes) and F_5_ (4 genotypes) generations (S3 Table). Furthermore, we identified all these multi-flowering lines in our breeding material in which the number of plants grown based on the phenotype was around 300 to 600 in different segregating generations. Thus, our data indicate the polygenic nature of this complex trait, which is again very much influenced by the environmental conditions as also explained in Fig 4. The multi-flowering trait in garden pea was observed to be expressed only on some but not every flowering node of all the multi-flowering genotypes known, including this study (Table 1). This clearly means that the expression of this trait is highly influenced by environmental factors, especially temperature. Furthermore, the multi-flowering trait was found attaining stabilization only during later generations (F_4_ onward) under some specific genetic combinations, indicating the possibility of accumulation of some alleles, giving rise to the expression of three to five flowers per raceme.

Similarly, an increase in the mean number of flowers per raceme has been reported with the accumulation of both dominant alleles [42] and recessive alleles [43]. In addition, the single recessive gene *fn* (*flower number*) controlling the multi-flowering trait has been reported by Vilmorin [22] and Wellensiek [44] in *Pisum*. Recently, the dominant epistasis interaction (13:3) causing in multi-flowering axillary raceme development in garden pea plants has been reported [9]. All these reports indicate the complex genetic expression of this trait. Since various stable and novel multi-flowering genotypes of different backgrounds have been developed by recent biotechnological tools, it is possible to find the gene(s)/QTLs regulating the multi-flowering mechanism(s) in these genotypes.

#### Role of environmental factors in the expression of the multi-flowering trait in *Pisum*

**Fig 5:**
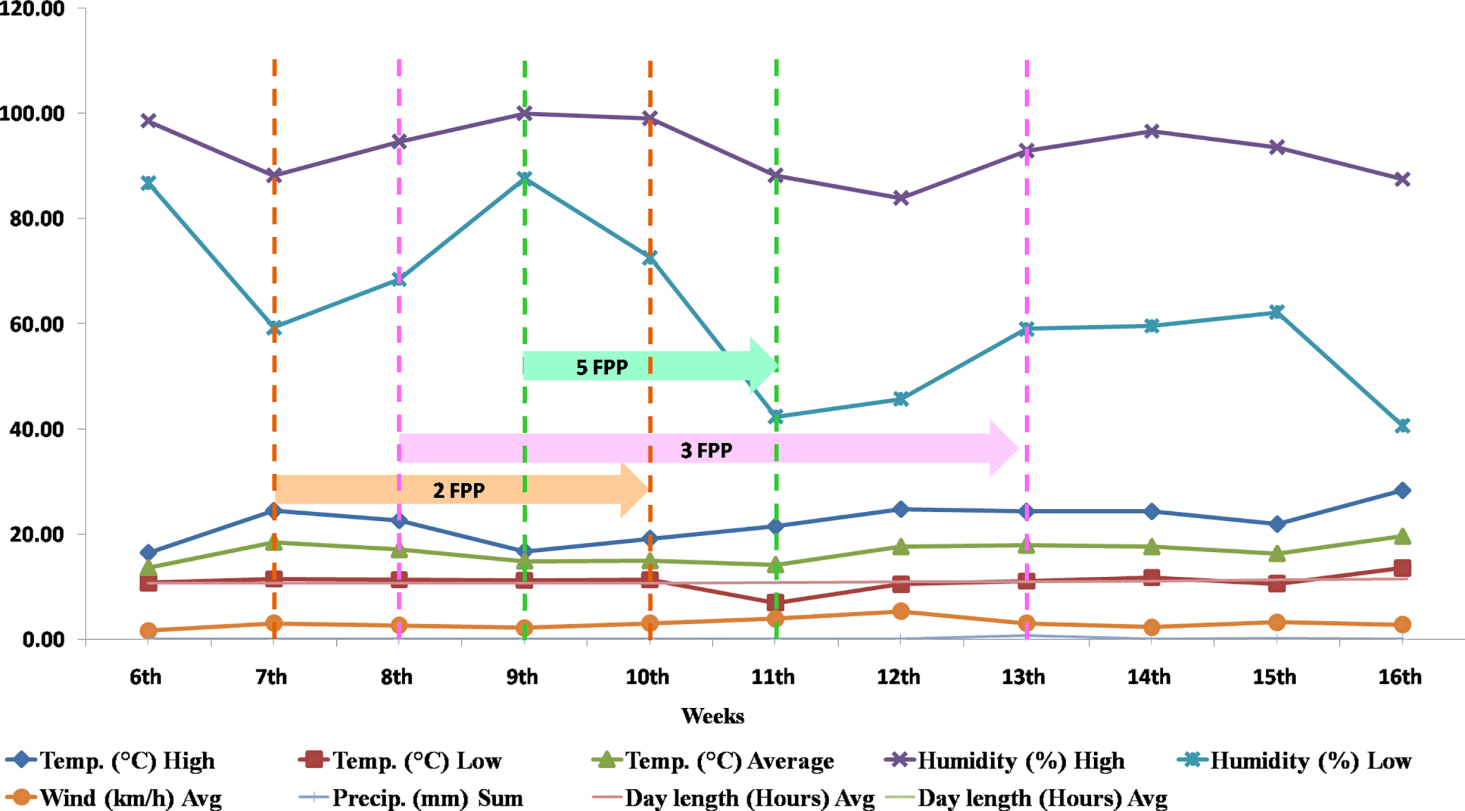
Comparison of various weather parameters with the multi-flowering trait in new pea genotypes. FPP: Flower per peduncle.

Even within the same genetic background, the variation in the flower numbers per peduncle has been recorded, which starts with two flowers per peduncle at the lower nodes, followed by three to five flowers per peduncle in middle, and then again one to two flower per peduncle toward the upper node (Fig 3). This means that in addition to genetic control, various growing conditions also regulate the number of flowers per I_2,_ which is a flower-bearing lateral raceme [15, 32,45]. To get further insight, three-year weather data (2015–16, 2016–17 and 2017–18) were critically analyzed for the possible role of weather in influencing the multi-flowering trait in the garden pea (S3–S5 Figs). Among seven multi-flowered lines, five (VRP–500, VRPM–501, VRPM–502, VRPM–901–3, and VRPM–901–5) showed flowering during seventh to eighth week after sowing (WAS), whereas VRPM–503 and VRPSeL–1 flowered during eighth to ninth week, which is late by one week (Table 4). During this period, the mean ambient temperatures were 15.2°C, 16.8°C and 13.6°C for the years 2015–16 and 2016–17 and 2017–18, respectively (Table 4; S3–S5 Figs; Fig 5).

**Table 4:** Sequential appearance and termination details of flowering in the new multi-flowering garden pea genotypes.

**Year**		**2015–16 (WAS)**						
**Number of Flowers per peduncle**		**VRP–500**	**VRPM–501**	**VRPM–502**	**VRPM–503**	**VRPM–901–3**	**VRPSeL–1**	**VRPM–901–5**
**2 Flowers**	**Appearance**	7^th^	7^th^	8^th^	9^th^	8^th^	9^th^	8^th^
	**Termination**	8^th^	8^th^	9^th^	10^th^	9^th^	10^th^	9^th^
**3 Flowers**	**Appearance**	8^th^	8^th^	9^th^	10^th^	9^th^	10^th^	9^th^
	**Termination**	11^th^	12^th^	12^th^	11^th^	12^th^	11^th^	12^th^
**5 Flowers**	**Appearance**	**-**	**-**	**-**	**-**	**-**	**-**	12^th^
	**Termination**	**-**	**-**	**-**	**-**	**-**	**-**	12^th^
**Overall flower termination**		13^th^	13^th^	14^th^	14^th^	16^th^	15^th^	16^th^
**Year**		**2016–17 (WAS)**						
**2 Flowers**	**Appearance**	7^th^	7^th^	7^th^	9^th^	7^th^	9^th^	7^th^
	**Termination**	9^th^	8^th^	8^th^	10^th^	8^th^	10^th^	8^th^
**3 Flowers**	**Appearance**	9^th^	8^th^	8^th^	10^th^	8^th^	10^th^	8^th^
	**Termination**	11^th^	12^th^	12^th^	11^th^	13^th^	12^th^	13^th^
**5 Flowers**	**Appearance**	**-**	**-**	**-**	**-**	**-**	**-**	9^th^
	**Termination**	**-**	**-**	**-**	**-**	**-**	**-**	11^th^
**Overall flower termination**		14^th^	13^th^	14^th^	14^th^	15^th^	14^th^	15^th^
**Year**		**2017–18 (WAS)**						
**2 Flowers**	**Appearance**	7^th^	7^th^	7^th^	8^th^	7^th^	9^th^	7^th^
	**Termination**	8th	8^th^	8^th^	9^th^	8^th^	10^th^	8^th^
**3 Flowers**	**Appearance**	8^th^	8^th^	8^th^	9^th^	9^th^	10^th^	8^th^
	**Termination**	10^th^	10^th^	11^th^	10^th^	12^th^	11^th^	12^th^
**5 Flowers**	**Appearance**	-	-	-	-	-	-	9^th^
	**Termination**	-	-	-	-	-	-	11^th^
**Overall flower termination**		13^th^	13^th^	14^th^	13^th^	15^th^	15^th^	15^th^

Where, WAS: Week after sowing.

Three flowers per peduncle appeared from eighth to tenth WAS, when mean temperatures were 15.9°C, 15.6°C and 12.7°C during winter seasons of 2015–16, 2016–17 and 2017–18, respectively (S3–S5 Figs). However, five-flowering racemes appeared during 12^th^ WAS when mean temperature was 13.6°C (2015–16), 09^th^ to 11^th^ WAS when the mean temperature was 14.6°C (2016–17) and from 09^th^ to 10^th^ WAS when the mean temperature was 12.9°C (2017–18), respectively. Interestingly, these weeks coincided with the low temperature of the seasons at Varanasi location for these three years. Thus, the narrow range of the expression of three to five flowers per peduncle appears very strongly influenced by the specific temperature range required by the apical bud. Although, it do require further detailed analysis under temperature controlled conditions. Moreover, lower light intensity (60 J–1s–2) and a temperature of around 20°C is known to increase the mean number of flowers per racemes in garden pea [15]. In addition, genotypic background and pea-growing locations are also known to cause variations in the flower number [30, 31].

### PC–531: A common parent giving multi-flowering phenotype in different combinations

For the progenies of three crosses, namely VRP–5×PC–531 (VRP–500), PC–531×Pusa Pragati (VRPM–501), and VL–8×PC–531 (VRPM–901–3), which expresses the multi-flowering trait, the genotype PC–531 was common either as a male or female parent. This led us to critically observe all the crosses of our ongoing breeding program having PC–531 as one parent in various generations. To our surprise, multi-flowering at some peduncle (although not stable) was observed in the segregating progenies of seven more crosses involving PC–531 as one parent, such as VL–3×PC–531 (F_3_), PC–531×NDVP–250 (F_4_), PC–531×Azad Pea–3 (F_4_), PC–531×PMR–53 (F_4_), PC–531×VRPMR–10 (F_4_), PC–531×VRPE–25 (F_4_), and PC–531×DARL–404 (F_4_). This indicates that the genotype PC–531 may possesses some genes of additive nature, as this trait got expressed not before F_4_ generation. However, it needs further confirmation using bi-parental cross of this line with other single-flower or two-flower lines. All the parents used to get the multi-flowering phenotype in our breeding program are either single- or double-flowering per peduncle-type genotypes. Such types of multi-flowering progenies from parents bearing single flowers were also identified in garden pea by Wellensiek [23]. Similarly, the inter-specific cross of chickpea, ICC–5783 (*Cicer arietinum*) × ICCW–9 (*C*. *reticulatum*), expressed three to nine flowers per peduncle [19].

### Integration of various multi-flowering models and linkage between multi-flowering and delayed maturity in garden pea

Based on physiological and mutation analyses, many gene models summarizing the regulation of flowering-time and flower-number per peduncle in *Pisum* have been proposed by various workers [4, 46, 47]. Vernalization and photoperiod are reported affecting both flowering time [4, 46], and variation in the first node of flower initiation in pea, which are found dependent on the combination of five major flowering loci, namely *SN* (*Sterile Nodes*), *LF* (*Late Flowering*), *PPD* (*Photoperiod*), *HR* (*High Response to photoperiod*), and *E* (*Early*) present in any pea genotype [36,48,49]. Further, three flowering loci namely, *Sn*, *Dne* (Day neutral), and *Hr*, was reported controlling multi-flowering trait [50], which along with *Lf* allele determines the actual floral node number in pea. Functionally, *Sn* [44, 51] and *Dne* [52] loci are known to confer photoperiod response, whereas *Hr* can prolong the expression of *Sn* and *Dne* genes. In another model, Murfet [32] proposed same genes (*SN*, *DNE*, *HR*, and *LF*) for explaining late flowering in pea. In addition, Lamprecht [24] reported two polymeric genes named *Flower Number* (*FN* and *FNA*) that in *FNFNA*, *fnFNA* or *FNfna*, and *fnfna* combinations produces one, two, and many-flowered I_2_s, respectively. Even a single gene, *Neptune*, having two recessive multi-flowering alleles, *nep–1* and *nep–2*, can produce three and more flowers per I_2_ [45].

The isolated reports of different gene models [24, 32, 45] explaining flowering-time and multi-flowering expression in pea was found very complicated and confusing. Thus, we have comprehensively integrated these isolated gene models explaining both multi-flowering and late-flowering trait in garden-pea (Fig 4). Since, both late-flowering and multi-flowering traits seem very tightly linked, we observed that same model was found working for multi-flowering traits too (Fig 4). On the similar note, all the multi-flowering genotypes identified in the present investigation are also of either mid or late maturity groups. However, the reverse is not true, depicting genotypic differences for the multi-flowering trait. This again confirms the assumption that multi-flowering genes are very tightly linked with late-flowering genes [16, 24]. However, this demands further in-depth investigation at genetic level.

Further, the *Lf* locus was known for the determination of minimum length of the vegetative period and the interval between openings of flowers at consecutive nodes [32, 50]. In this study, all the genotypes, namely, VRP–500, VRPE–501, VRPE–502, VRPE–503, and VRPM–901–3 and VRPSeL–1, the first multi-flowering peduncle bearing three flowers, appeared to vary from 14^th^ to 16^th^ node, while in the five flowers per peduncle genotype (VRPM–901–5), its average was 21.93, suggesting allelic variation for the *LF* locus in these plants.

## Conclusions

The multi-flowering garden pea genotypes identified in this study are exclusively expressed in either mid-flowering or late-flowering genotypes. Further, the role of environmental factors like temperature (11–20°C) was also fund regulating the expression of this trait (Fig 4). The sequential appearance of two or three flowers per peduncle at the lower flowering node, followed by the five-flowering peduncle in the middle flowering nodes, and again three-, two-, and one-flower peduncles toward the upper nodes, appears associated with a specific temperature range. In the era of genomics and transcriptomics, the differential expressions of multi-flowering traits need confirmation and identification of genes involved in regulating multi-flowering traits using transcriptomic approaches.

Interestingly, a common parent PC–531 was identified, which might be having some genes of additive nature, as this trait got expressed not before F_4_ generation. Further, most of our genotypes derived from this parent expressed multi-flowering trait. However, this needs further confirmation using genetic studies involving various stable multi-flowering and single-flowering genotypes through an intensive crossing program. Thus, different multi-flowering genotypes, reported in this study including PC–531, seem prospective parents for any breeding program aimed at incorporating multi-flowering trait in garden-pea. Preliminary observations do indicated significant yield advantage in the multi-flowering genotypes identified in this study over single and double-flowering genotypes. However, further multi-location field trials of these multi-flowering lines are required to conclusively demonstrate the importance of this trait in improving garden pea yield.

## Supporting information

S1 Fig Experimental methodology involving different parents and crosses to develop multi-flower genotypes in garden peas. FPP: Flower per Peduncles.
(PNG)

S2 Fig Weather parameters recorded during winter-season of the year 2014–15 at ICAR-IIVR, Varanasi, India. (PNG)

S3 Fig Weather parameters recorded during winter-season of the year 2015–16 at ICAR-IIVR, Varanasi, India. (PNG)

S4 Fig Weather parameters recorded during winter-season of the year 2016–17 at ICAR-IIVR, Varanasi, India. (TIF)

S5 Fig Weather parameters recorded during winter-season of the year 2017–18 at ICAR-IIVR, Varanasi, India. (TIFF)

S6 Fig Variations in the thickness of the peduncle in VRPM-901-5 (5 flowers per peduncle) and VRPSeL-1 (3 flowers per peduncle) genotypes. (TIF)

S1 Table Characterization of VRPM-901-5 (bearing 5 flowers per peduncle) for inflorescence related trait. (DOCX)

S2 Table Analysis of variance (ANOVA) for yield and related traits in multi-flowering genotypes and double flowered cultivars. (DOCX)

S3 Table Details of multi-flowering genotypes, their pedigree, and generation in which multi-flowering trait was first recorded. (DOCX)
