# Supplementary figures and images for: Development and characterization of penta-flowering and triple-flowering genotypes in garden pea (Pisum sativum L. var. hortense)
Source: PLoS One. 2018 Jul 30;13(7):e0201235. doi: 10.1371/journal.pone.0201235 (PMC6066227; doi:10.1371/journal.pone.0201235)

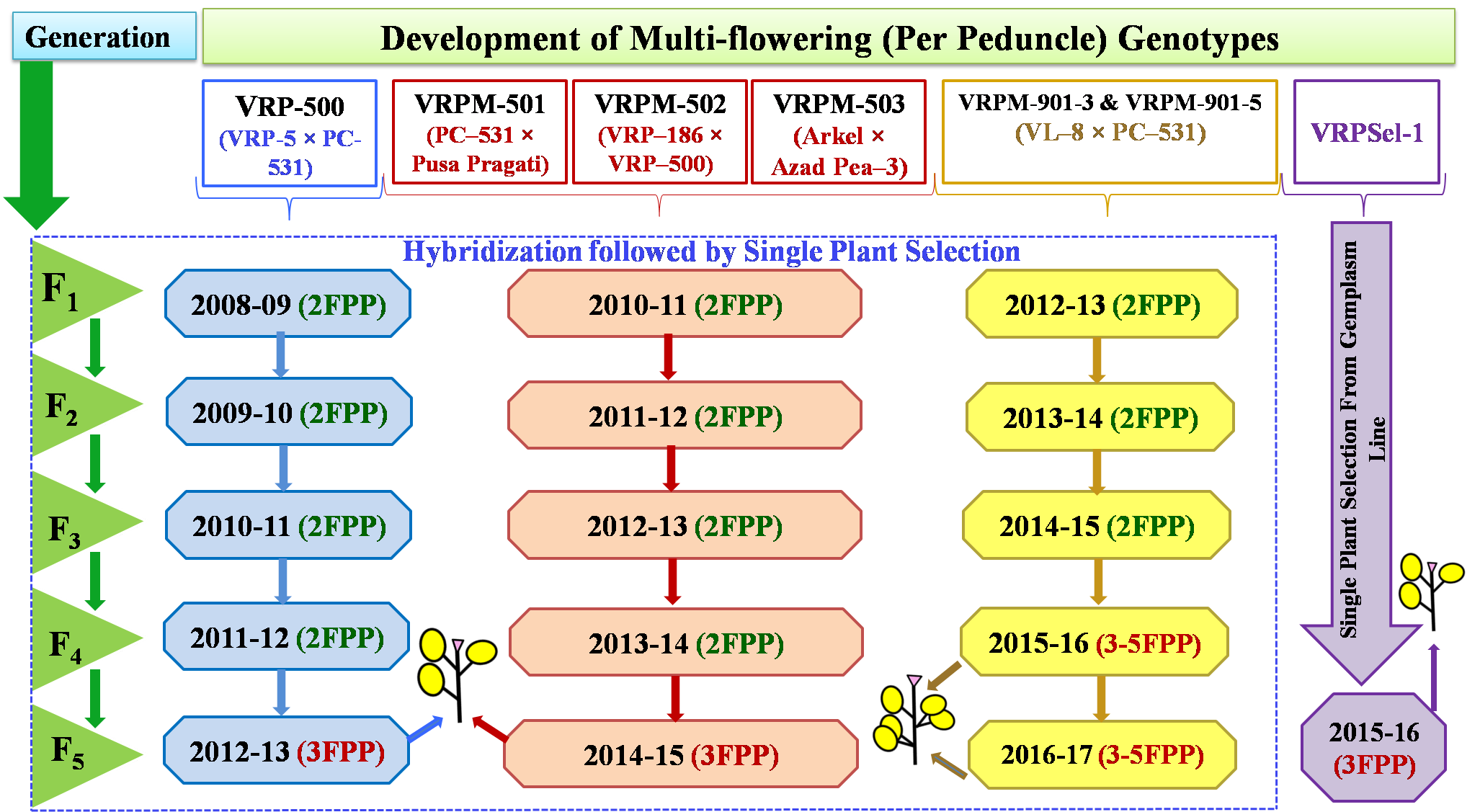

Supplement: S1 Fig — FPP: Flower per Peduncles. (PNG) [file pone.0201235.s001.png]

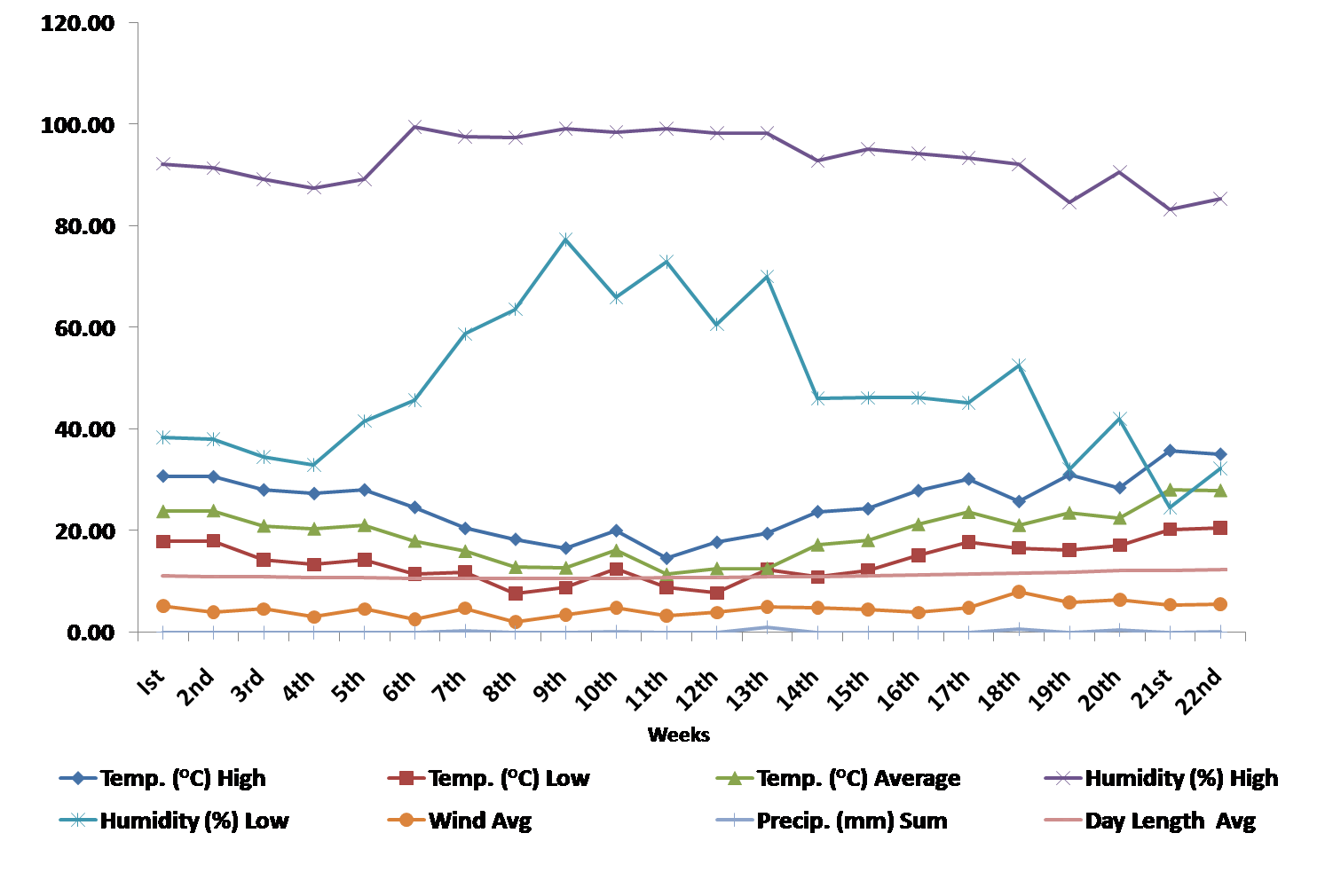

Supplement: S2 Fig — (PNG) [file pone.0201235.s002.png]

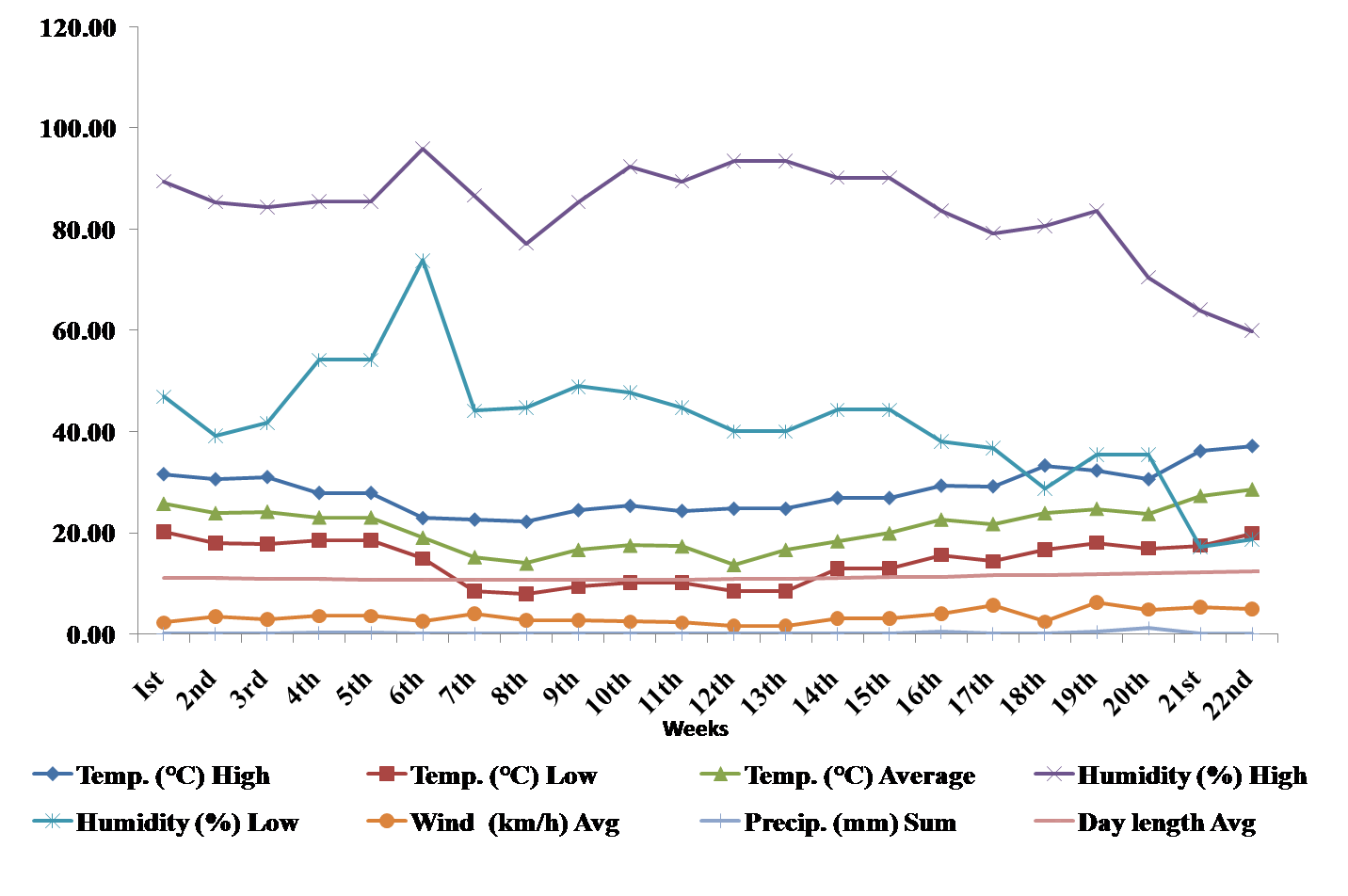

Supplement: S3 Fig — (PNG) [file pone.0201235.s003.png]

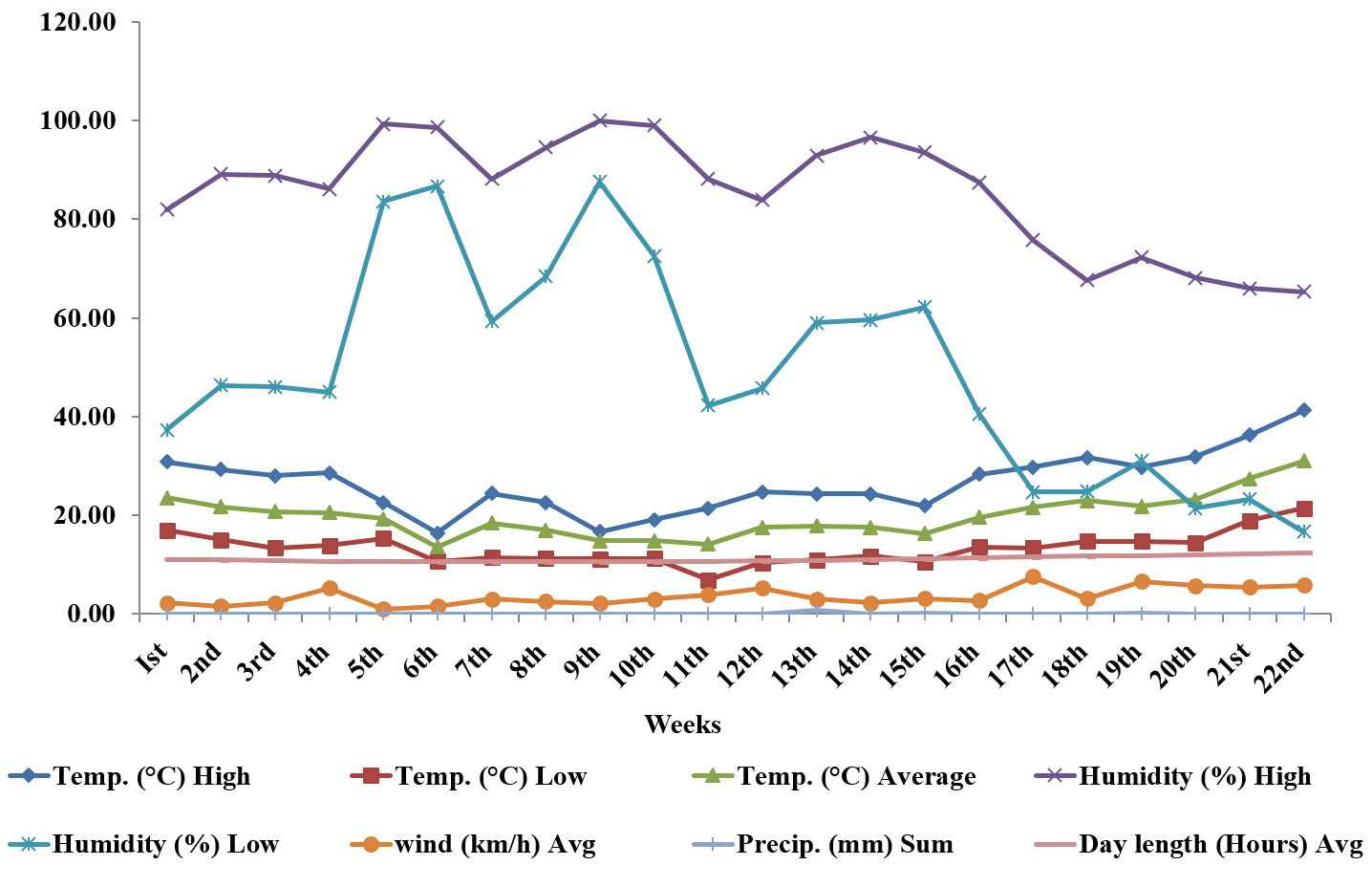

Supplement: S4 Fig — (TIF) [file pone.0201235.s004.tif]

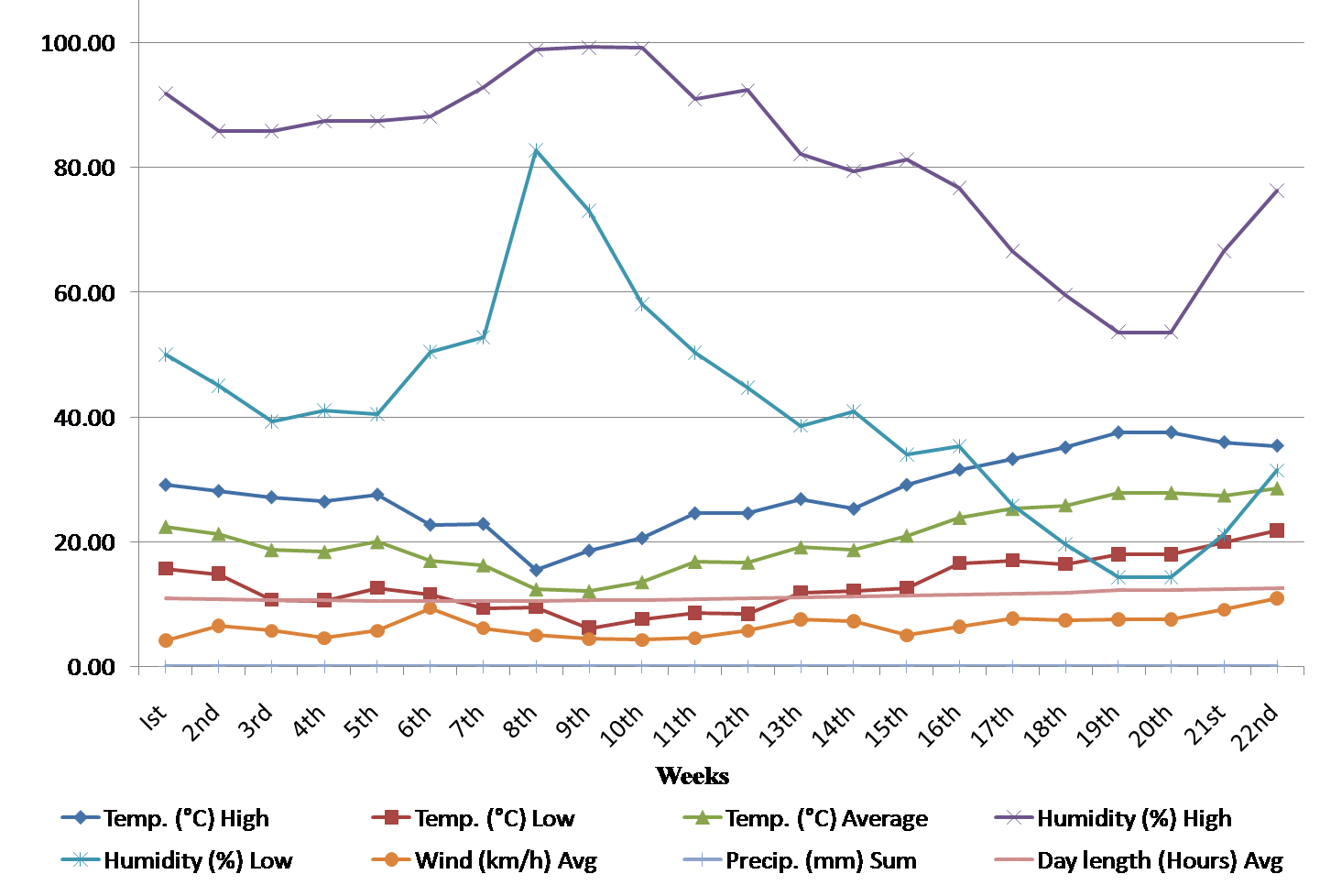

Supplement: S5 Fig — (TIFF) [file pone.0201235.s005.TIFF]

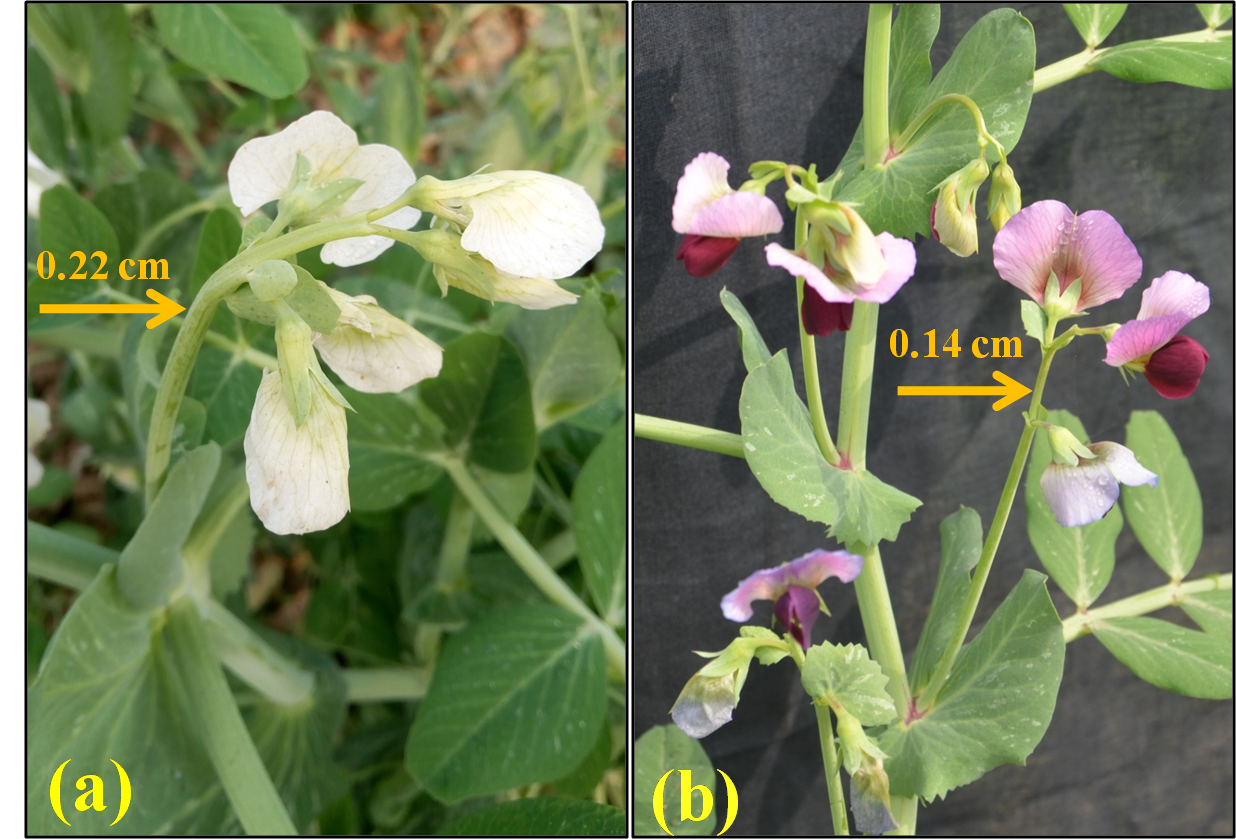

Supplement: S6 Fig — (TIF) [file pone.0201235.s006.tif]
