# Supplementary material for: Development and characterization of penta-flowering and triple-flowering genotypes in garden pea (Pisum sativum L. var. hortense)
Source: PLoS One. 2018 Jul 30;13(7):e0201235. doi: 10.1371/journal.pone.0201235 (PMC6066227; doi:10.1371/journal.pone.0201235)
Supplement: S1 Table — (DOCX) [file pone.0201235.s007.docx]

**S1 Table. Characterization of VRPM-901-5 (bearing 5 flowers per peduncle) for inflorescence related trait.**

| **^1^Traits/**  **Plant No.** | **FFN** | **NON**  **producing 1**  **flower** | **NON**  **producing 2 flower** | **NON**  **producing 3 flower** | **NON**  **producing 4 flower** | **NON**  **producing 5 flower** | **Node bearing 5 flowered raceme** | **TFP** | **TRN** |
| --- | --- | --- | --- | --- | --- | --- | --- | --- | --- |
| 1 | 20 | 5 | 7 | 16 | 0 | 1 | 21 | 72 | 29 |
| 2 | 20 | 1 | 3 | 7 | 0 | 1 | 22 | 33 | 12 |
| 3 | 20 | 1 | 10 | 7 | 0 | 2 | 22,26 | 52 | 20 |
| 4 | 19 | 1 | 1 | 6 | 0 | 2 | 23, 25 | 31 | 10 |
| 5 | 21 | 0 | 10 | 3 | 0 | 5 | 23, 25, 27, 29, 30 | 54 | 18 |
| 6 | 21 | 0 | 8 | 22 | 0 | 2 | 22, 24 | 92 | 32 |
| 7 | 22 | 1 | 8 | 14 | 1 | 1 | 24 | 68 | 25 |
| 8 | 23 | 3 | 4 | 5 | 0 | 1 | 23 | 31 | 13 |
| 9 | 23 | 0 | 6 | 2 | 0 | 1 | 23 | 23 | 9 |
| 10 | 23 | 1 | 4 | 2 | 0 | 1 | 23 | 20 | 8 |
| 11 | 23 | 0 | 9 | 14 | 0 | 1 | 23 | 65 | 24 |
| 12 | 24 | 2 | 11 | 3 | 0 | 1 | 23 | 38 | 17 |
| 13 | 23 | 2 | 12 | 2 | 0 | 1 | 23 | 37 | 17 |
| 14 | 24 | 7 | 10 | 6 | 0 | 2 | 26, 26 ^(sb)^ | 55 | 25 |
| 15 | 23 | 0 | 4 | 19 | 0 | 2 | 26, 28 | 75 | 25 |
| **Total** | **-** | **24** | **107** | **128** | **1** | **24** | **24** | **746** | **284** |
| **Mean±SE** | **21.93±0.40** | **1.60±0.52** | **7.13±0.85** | **8.53±1.72** | **0.07±0.07** | **1.60±0.27** | **-** | **49.73±5.53** | **18.93±1.95** |
| **CV** | **7.41** | **126.77** | **46.45** | **78.35** | **387.30** | **65.97** | **-** | **43.05** | **39.90** |

**^1^FFN**: First flowering node; **NON**: Number of Node; **TFP**: Total flower produced; **TRN**: Total reproductive nodes; **sb**: Secondary branch
