# Supplementary material for: Development and characterization of penta-flowering and triple-flowering genotypes in garden pea (Pisum sativum L. var. hortense)
Source: PLoS One. 2018 Jul 30;13(7):e0201235. doi: 10.1371/journal.pone.0201235 (PMC6066227; doi:10.1371/journal.pone.0201235)
Supplement: S2 Table — (DOCX) [file pone.0201235.s008.docx]

**S2 Table. Analysis of variance (ANOVA) for yield and related traits in multi-flowering genotypes and double flowered cultivars.**

| **Traits** | **Source of variation** | | | |
| --- | --- | --- | --- | --- |
|  | **Mean squares** | | | |
|  |  | **Replication** | **Treatments** | **Error** |
|  | **Df** | 2 | 8 | 16 |
| Days to first flowering |  | 23.88 | 173.75^**^ | 3.42 |
| Appearance of first multi-flowering node |  | 5.58 | 160.64^**^ | 1.60 |
| Branches per plant |  | 0.032 | 0.35^**^ | 0.036 |
| Peduncle length (cm) |  | 0.57 | 18.88^**^ | 0.12 |
| Peduncle diameter (cm) |  | 0.0007 | 0.0028** | 0.0006 |
| Pod length (cm) |  | 0.61 | 5.74^**^ | 0.59 |
| Pod width (cm) |  | 0.001 | 0.187^**^ | 0.018 |
| Average pod weight (g) |  | 0.737 | 2.93^**^ | 0.19 |
| Pods per plant |  | 33.16 | 89.36^**^ | 9.19 |
| Seeds per pod |  | 0.095 | 2.047^**^ | 0.30 |
| Plant height (cm) |  | 163.62 | 1324.68^**^ | 93.54 |
| Total flower produced |  | 33.18 | 238.47^**^ | 9.33 |
| Yield per plant (g) |  | 1573.76 | 2796.96^**^ | 207.51 |
| Percent Conversion to multi-pods |  | 176.44 | 3159.54^**^ | 26.61 |

Significant at **P ≤ 0.01
