# Supplementary material for: Development and characterization of penta-flowering and triple-flowering genotypes in garden pea (Pisum sativum L. var. hortense)
Source: PLoS One. 2018 Jul 30;13(7):e0201235. doi: 10.1371/journal.pone.0201235 (PMC6066227; doi:10.1371/journal.pone.0201235)
Supplement: S3 Table — (DOCX) [file pone.0201235.s009.docx]

**S3 Table. Details of multi-flowering genotypes, their pedigree, and generation in which multi-flowering trait was first recorded.**

| **S. No.** | **Multi-flowering**  **genotype** | **Pedigree** | **Generation in which multi-flowering was first recorded** |
| --- | --- | --- | --- |
|  |  |  |  |
|  | VRPM–501 | PC–531 × PusaPragati | F_5_ |
|  | VRPM–502 | VRP–186 × VRP–500 | F_5_ |
|  | VRPM–503 | Arkel × Azad Pea–3 | F_5_ |
|  | VRPM–901–3 | VL–8 × PC–531 | F_5_ |
|  | VRPM–901–5 | VL–8 × PC–531 | F_4_ |
|  | VRPM–504 | VL–3 × PC–531 | F_4_ |
|  | VRPM–505 | PC–531 × NDVP–250 | F_4_ |
|  | VRPM–506 | PC–531 × AzadPea–3 | F_4_ |
|  | VRPM–507 | PC–531 × PMR–53 | F_4_ |
|  | VRPM–508 | PC–531 × VRPMR–10 | F_4_ |
|  | VRPM–509 | PC–531 × VRPE–25 | F_4_ |
|  | VRPM–510 | PC–531 × DARL–404 | F_4_ |
|  | VRPSeL–1 | *SiharaLocaL–1* (Selection) | Natural mutant |
